# Impact of Suture Conchopexy on Olfaction and the Risk of Middle Turbinate Lateralization

**DOI:** 10.7759/cureus.5814

**Published:** 2019-10-01

**Authors:** Ibrahim Sumaily, Ibrahim Alarifi, Labeb M Sailan, Saad Alsaleh, Mohammad Aloulah

**Affiliations:** 1 Otorhinolaryngology, King Abdulaziz University Hospital, Riyadh, SAU; 2 Otorhinolaryngology, King Saud University, Riyadh, SAU

**Keywords:** keywords: conchopexy, middle turbinate, lateralization, endoscopic, sinus

## Abstract

Introduction: Middle turbinate (MT) lateralization is one of the common causes of endoscopic sinus surgery (ESS) failure and often necessitate revision surgery. To avoid this sequala, surgeons have attempted several methods to keep the MT medialized. One such method is conchopexy. However, the impact of this procedure on olfaction remains unclear.

Method: A retrospective cohort study was conducted to compare the subjective olfaction outcome of ESS in patients for whom conchopexy was performed and in controls where a spacer was applied in the middle meatus. Also, the risk of lateralization in both techniques was compared. In addition, other factors related to the outcome of olfaction, such as age, gender, type of chronic rhinosinusitis (CRS), and partial resection of the MT, were assessed.

Results: Out of 299 patients with CRS who underwent ESS, 134 met our inclusion criteria. In total, 62.7% were male and 37.3% were female, and their mean age was 37.4 years. Sixty-one patients (cases) underwent conchopexy, and 73 patients (controls) underwent insertion of a middle meatus spacer. None of the subjects in both groups developed anosmia or hyposmia as a complication. The improvement of olfaction was almost equal in both groups (for anosmia: 92.9% in cases vs. 87.5% in control; for hyposmia 87.1% in cases vs. 89.7% in control). In patients with anosmia, the improvement of olfaction was lower when the MT was partially resected (71.4% vs. 95.7%); whereas, in patients with hyposmia, the improvement was not significantly different (87% vs. 93.8% when the MT was partially resected). The improvement of olfaction was higher in patients with allergic fungal sinusitis (AFS) and CRS with nasal polyps (CRSwNP) than in those with CRS without nasal polyps (CRSsNP). The MT lateralization was almost equal in both groups (9.0% in cases vs. 9.6% in controls).

Conclusion: Conchopexy does not affect olfaction subjectively. The improvement of olfaction is related more to the underlying disease, i.e., less improvement occurs in cases of CRSsNP. The risk of lateralization is equal with either conchopexy or middle meatus spacer.

## Introduction

The middle turbinate (MT) is an important surgical landmark in functional endoscopic sinus surgery (FESS) [1]. Postoperatively, lateralization of the MT with scarring and obstruction of the middle meatus after endoscopic ethmoidectomy has accounted for a high percentage of postoperative complications [2-5]. However, some published reports have mentioned that it is the most common complication of endoscopic sinus surgery and often necessitate a revision surgery [5-6]. Turbinate medialization techniques have gained popularity, as its main aim is to prevent turbinate lateralization [3,7]. Theoretically, adhesions between the septum and MT prevent lateralization but may compromise airflow to the olfactory neuroepithelium and affect the sense of olfaction [3].

MT medialization without resection is an approach designed to both preserve the MT and prevent lateralization, which may cause obstruction of the airflow in the ethmoid, maxillary, and frontal sinuses after endoscopic sinus surgery [8].

Numerous methods have been used to assist in the avoidance of this complication including the use of packing in the ethmoid sinus as a "spacer", creation of synechia between the MT and septum (bolgerization), and suture medialization of the MT to the septum (conchopexy) [5,9]. While the conchopexy is an effective technique, because the olfactory groove lies superior in the groove between the MT and septum, concerns have been raised as to the effect of this maneuver on olfaction [9]. The long-term results of these techniques are lacking [10]. The conchopexy technique has been criticized for being technically difficult and time-consuming, mainly due to the need to tie a knot within the nasal cavity; therefore, another technique called non-knot conchopexy is applied by using double through and through sutures without applying a knot to the suture ends [11].

In our center, either the conchopexy or middle meatus spacer technique is used for almost every patient. In this study, we aimed to assess the risk for olfaction loss in patients who undergo conchopexy and middle meatus packing and to determine the incidence of MT lateralization in these two groups.

## Materials and methods

This retrospective cohort study was conducted in our tertiary hospital. After obtaining the approval of the institutional review board to conduct this study, we reviewed all of the patients who underwent endoscopic sinus surgery with either middle meatus packing or suture conchopexy for chronic rhinosinusitis (CRS) and followed up for at least six months. We excluded those who underwent revision surgery, those who underwent complete resection of the MT, those with benign or malignant tumors and those with incomplete records or missing data. We reviewed olfaction status pre- and post-operatively, and the position of the MT postoperatively. Other variables studied include age, gender, type of CRS, surgical procedures done for the middle turbinate, septoplasty, inferior turbinate intervention, use of steroids postoperatively, patency of the nasal airways pre- and postoperatively, patency of the sinus ostia postoperatively. Subjects were divided into two groups; the first group is those who underwent endoscopic sinus surgery with their MTs kept medialized with suture conchopexy to the septum (cases), and the second group are those whose MTs were kept medialized through the insertion of a spacer in the middle meatus, NasoPore (controls). We focused mainly on the subjective olfaction outcome in these two groups and the factors associated with this outcome according to their follow up assessment records between six months to one year. The preoperative status of olfaction was normal olfaction, hyposmia, or anosmia. The postoperative status of olfaction was normal olfaction as preoperative status, improved hyposmia or anosmia, non-improved hyposmia or anosmia, or new onset of hyposmia or anosmia. This assessment is done at the follow-up visit between six and 12 months postoperatively. We used SPSS software, version 22 (SPSS Inc., Chicago, IL) for data analysis.

## Results

In total, 299 patients with CRS who underwent ESS between January 2015 and December 2017 were enrolled in this study. Of them, 134 met our inclusion criteria. The other 165 patients were excluded for the following reasons: 78 patients had undergone surgery previously, 22 had tumors, 16 had undergone complete resection of the MT, and 49 had incomplete records. Patient age ranged between 14 and 80 years, and the mean age was 37.4 years, with a standard deviation of 13.8 years. In total, 62.7% were male and 37.3% were female. Preoperatively, 87.3% had partial nasal obstruction, 9.0% had complete nasal obstruction, and the remaining 3.7% had no nasal obstruction. Of those patients with nasal obstruction, 79.9% had bilateral nasal obstruction. Preoperatively, 52.2% had hyposmia, 22.4% had anosmia, and the rest (25.4%) had normal olfaction. There was no gender difference in the preoperative status of olfaction. All those with no nasal obstruction were having normal olfaction. The most prevalent disease type was CRS with polyps (CRSwNP) (64.9%), followed by CRS without nasal polyps (CRSsNP) (27.6%) and allergic fungal sinusitis (AFS) (7.5%). The prevalence of CRSwNP was higher in males, whereas the prevalence of CRSsNP and AFS were higher in females. The prevalence of AFS in females was four times that in males. In total, 98.5% had bilateral disease. All those with complete obstruction had CRSwNP, and bilateral nasal obstruction was higher in patients with CRSwNP. In addition, patients with CRSwNP always had bilateral disease. Olfaction was less affected in patients with CRSsNP (43.2%) as seen in Table 1. The MTs were partially resected in 22.4% of the cases either because of polypoidal mucosa or concha bullosa. The remaining patients did not undergo MT reduction.

Suture conchopexy was done for 61 patients (cases), and the middle meatus spacer was used for 73 patients (controls). None of the subjects in both groups, who have normal olfaction preoperatively (34 candidates), developed new-onset anosmia or hyposmia as a complication after surgery. Overall olfaction improvement occurred in 89%. The conchopexy showed no negative impact on the olfaction improvement (88.9% vs. 89.1% in the middle meatus group, P-value= 0.975). This was not statistically affected by the age or the gender of the candidates. In patients with anosmia, the improvement of olfaction was lower when the MT was partially resected (71.4% vs. 95.7%), whereas, in patients with hyposmia, the improvement was slightly higher when the MT was partially resected (87% vs. 93.8%) (Tables 1-2). The improvement of olfaction was higher in patients with AFS than in patients with CRSwNP or those with CRSsNP (100%, 89.5%, and 81%, respectively). Systemic steroid administration postoperatively was associated with higher improvement of olfaction among subjects, but not statistically significant (p-value 0.269).

**Table 1:** Comparison between the suture conchopexy and middle meatus spacer groups MT: Middle turbinate, CRSwNP: Chronic rhinosinusitis with nasal polyposis, CRSsNP: Chronic rhinosinusitis without polyposis, AFS: Allergic fungal sinusitis.

Variable	Spacer	Conchopexy	P value
N	73	61	
Age	35.8 (+ 14.8)	39.4 (+ 12.2)	0.136
Gender (male)	53.4%	73.8%	0.015
Preoperative nasal obstruction (partial)	82.2%	93.4%	0.563
Preoperative hyposmia or anosmia	75.3%	73.8%	0.964
Diagnosis (CRSwNP, CRSsNP, AFRS)	57.5%, 34.2%, 8.2%	73.8%, 19.7, 6.6	0.102
Partial resection of the MT	26.0%	18.0%	0.272
Septoplasty	73.1%	66.7%	0.610
Inferior turbinate reduction	78.8%	61.1%	0.142
Olfaction improvement Postoperatively	89.1%	88.9%	0.975
Postoperative systemic steroid administration	60.3%	68.9%	0.306
Postoperative nasal obstruction (subjective)	4.1%	0.0%	0.111
Postoperative nasal airway (patent)	98.6%	100%	0.363
Middle turbinate lateralization	9.6%	9.0%	0.825

**Table 2:** Comparison between cases of improved olfaction postoperatively and non-improved olfaction postoperatively groups MT: Middle turbinate, CRSwNP: Chronic rhinosinusitis with nasal polyposis, CRSsNP: Chronic rhinosinusitis without polyposis, AFS: Allergic fungal sinusitis.

Variable	Improved olfaction	Not improved	P value
N	89	11	
Age	35.84 (+ 13.2)	41 (+ 12.57)	0.224
Gender (male)	61.8%	54.5%	0.646
Preoperative nasal obstruction (partial)	87.6%	90.9%	0.756
Preoperative olfaction status (hyposmia or anosmia)	100%	100%	1.000
Diagnosis (CRSwNP, CRSsNP, AFRS)	76.4%, 14.6%, 9%	72.7%, 27.3, 0.0	0.789
Partial resection of the MT	22.5%	27.3%	0.724
Septoplasty	73.5%	75%	0.951
Inferior turbinate reduction	73.5%	75%	0.951
Suture Conchopexy	44.9%	45.5%	0.975
Postoperative systemic steroid	78.7%	63.6%	0.269
Postoperative nasal obstruction (subjective)	0.0%	18.2%	0.001
Postoperative nasal airway (patent)	98.9%	100%	0.727
Middle turbinate lateralization	11.2%	9.1%	0.688

## Discussion

As part of endoscopic sinus surgery, the MT is almost always medialized to perform middle meatus antrostomy, ethmoidotomy, and frontal sinusotomy. The MT can lateralize postoperatively, and several techniques to prevent lateralization of the MT and its sequelae have been described and discussed in the literature, such as controlled synechia (bolgerization) [12], insertion of metallic clips between the head of the MT and septum [1], suture conchopexy [2], and synechia with bovine serum albumin tissue adhesive [8].

Medialization techniques are theoretically expected to interfere with olfaction. In our study, we did not note a significant difference in the subjective olfaction outcome between the suture conchopexy and middle meatus spacer groups, and this result is in line with the findings of the prospective controlled study by Friedman et al. that compared olfaction before and after MT medialization [3]. In addition, Dutton et al. who conducted a prospective non-controlled study of patients who underwent functional endoscopic sinus surgery with suture medialization of the MT reported a small but significant improvement in olfactory function after MT suture medialization to the septum compared with the preoperative status, as assessed using the University of Pennsylvania Smell Identification Test (UPSIT) [9]. Hegazy et al. who compared on 39 patients the olfaction outcome of non-intervention, bolgerization, and suture conchopexy groups reported no significant improvement of olfaction in all the three groups [13].

In our study, we found that the incidence of lateralization in those who underwent suture conchopexy was almost equal to that in patients who underwent insertion of a spacer in the middle meatus, and this number is higher than what Thorton RS mentioned in his retrospective study (9% vs. 1.67%) [2]. This number is also higher than the incidence of lateralization noted when using controlled synechia, as reported by Friedman et al. who used the microdebrider to induce synechia between the MT and septum [6]. The incidence of lateralization in the present study is slightly lower than what Hewitt and Orlandi reported through their retrospective study; in their study, adhesions between the MT and lateral nasal wall developed in 10.8%, but most of these adhesions were easily released in the clinic [7]. In a prospective study by Dutton et al. on suture conchopexy in endoscopic sinus surgery cases, postoperative endoscopic examination revealed that lateralization of the MT was a rare complication after suture medialization of the MT [9]. But in this study no control group to compare.

There were no specific olfaction outcome tool results available in the patient records. Actually, there is no validated Arabic version of UPSIT. Although we used the subjective assessment of the olfaction status pre and postoperatively, it is very unlikely to fall in the estimation bias of the visual analogue scale, which can be affected by the outcome of the other symptoms. Also, for this reason, we excluded 49 of our candidates because of the incomplete or unclear records of their olfaction status either pre- or postoperatively.

The combination of both suture conchopexy and middle meatus spacer insertion was studied by Chen et al. in their prospective, randomized, blind controlled study; they found that the combination was associated with a significantly lower incidence of lateralization as well as synechia formation between the MT and lateral nasal wall [14].

In addition, Hanna and Kilty compared the cost-effectiveness of suture medialization and middle meatus stenting in 60 cases. They found that the commercial stent use cost ranged from 8 to 83 times the price of the suture depending on the stent [15].

Regarding the risk for disease recurrence and the need for revision surgery, Bofares, in his controlled trial involving four groups, namely, the medialization only, partial MT resection, medialization with controlled synechia, and septo-turbinal suture groups, found that medialization only was associated with 49% recurrence of sinusitis, partial resection was associated with 12% recurrence, and in the other two groups, there was no recurrence [16].

Our study is limited by being retrospective study and by using subjective olfaction status, not the UPSIT. However, UPSIT is also a subjective test and can confuse between normosmia and hyposmia as it is affected by the educational and cultural background of the candidate as several studies showed. No previous study tackled both the olfaction and the risk of lateralization on the same candidates with such sample size comparing conchopexy to middle meatus spacer. Our study supports that the conchopexy, which is a very cost effective, is equivalent to the middle meatus spacer in preventing lateralization of the MTs and is not interfering with olfaction.

## Conclusions

Conchopexy does not affect the olfaction subjectively. Neither conchopexy nor middle meatus spacer insertion was associated with olfaction loss among our cases. The number of patients who underwent MT lateralization with conchopexy was equal to that of those who underwent middle meatus spacer insertion. The olfaction improvement is related more to the underlying disease, as less improvement occurs in those with CRSsNP. Further prospective studies on the long-term outcome of conchopexy, its cost-effectiveness, and its impact on olfaction and the nasal airway using validated outcome tools are recommended.
